# An artificial intelligence approach for selecting effective teacher communication strategies in autism education

**DOI:** 10.1038/s41539-021-00102-x

**Published:** 2021-09-01

**Authors:** Vasileios Lampos, Joseph Mintz, Xiao Qu

**Affiliations:** 1 Department of Computer Science, University College London, London, UK; 2 Institute of Education, University College London, London, UK

**Keywords:** Education, Human behaviour

## Abstract

Effective inclusive education is key in promoting the long-term outcomes of children with autism spectrum conditions (ASC). However, no concrete consensus exists to guide teacher-student interactions in the classroom. In this work, we explore the potential of artificial intelligence as an approach in autism education to assist teachers in effective practice in developing social and educational outcomes for children with ASC. We form a protocol to systematically capture such interactions, and conduct a statistical analysis to uncover basic patterns in the collected observations, including the longer-term effect of specific teacher communication strategies on student response. In addition, we deploy machine learning techniques to predict student response given the form of communication used by teachers under specific classroom conditions and in relation to specified student attributes. Our analysis, drawn on a sample of 5460 coded interactions between teachers and seven students, sheds light on the varying effectiveness of different communication strategies and demonstrates the potential of this approach in making a contribution to autism education.

## Introduction

Autism education has been a growing area of interest in recent years, as the observed prevalence of autism spectrum condition (ASC) among children has risen from an estimated 1 in 10000 in the 1960s^1^ to at least 1 in 100 today^2,3^. ASC is an umbrella term that describes neurodevelopmental conditions which are typically expressed in terms of impaired social interaction and communication abilities, and stereotypical or obsessive patterns of behaviour. Such impairments often have a significant impact on the individual’s social, educational, and employment experiences within the current societal norms^4^. As such, the long-term outcomes of young people with ASC are often poor, and are associated with significant difficulties in undertaking complex and longer-lasting social transactions, acting independently in the labour market, or fulfilling job requirements^5^.

The potential of artificial intelligence (AI) to drive developments in education is well-recognised^6,7^. Currently, most research efforts are based on data stored in learning management systems, as this type of analysis is more straightforward^8^. In this paper, we report on an innovative experiment that uses machine learning, a data-driven approach to AI, to model teacher-student interactions in the classroom. Our particular focus is on children with ASC. The application of communication strategies tailored to the specific needs of children with ASC can lead to improved outcomes for this group^9–11^, including effective participation in educational opportunities, improved social functioning, and longer-term achievement in employment and relationships^5,12,13^. We propose that machine learning may be one way to further the development of such ASC-specific strategies. To evaluate our hypothesis, we devised a protocol for recording real-time in-classroom data capturing interactions between teachers and primary school students with ASC, including contextual information. In total, we coded 5460 interactions between teachers and a cohort of seven students with fairly heterogeneous characteristics. We then developed a classifier for predicting the student’s response to particular communication strategies by the teachers, and used this to ultimately suggest the most appropriate ones.

Our review of the literature indicates that most work to date involving machine learning and autism has focused on the development of screening tools for diagnosis, developing classifiers that could perform an earlier and more accurate ASC diagnosis and evaluation^14–16^. No research so far has investigated the application of machine learning to real-time data capturing teacher communication strategies and student response, and hence this "in vivo” modelling approach for education and autism remains largely unexplored. Hence, our study provides some evidence to support the argument that AI methods could be used to tailor pedagogical strategies to better meet the needs of the students with ASC.

We focused on teacher communication strategies because communication is a key impairment in ASC^17,18^. Research in autism education has highlighted their importance in learning and developing social communication skills^19–21^. We considered different types of teacher communication strategies and in particular verbal communications, the use of gestures, physical prompts, visual representations (pictures) or physical objects, and the extent to which children with ASC responded to different strategies. These strategies are typically employed as part of the social communication, emotional regulation and transactional support (SCERTS) framework, a widely used comprehensive whole-school approach to communication development in autism education^22,23^.

In typical teacher-student interactions in the classroom most communication is verbal^24^. However, traditional verbal communication alone often does not work effectively with children with ASC^25,26^. There is a particular emphasis in the literature on the use of visual strategies for effective communication with children and young people with ASC^27,28^. For example, some studies have shown that interventions using pictures and symbols such as the Picture Exchange Communication System can improve communication in non-verbal children with ASC^29–32^. Multiple studies on the use of visual activity schedules over a period of 20 years (1993–2013) corroborate these findings^33^. The use of objects in developing communication skills in children with ASC has also been noted^30,34,35^. Objects may provide a mode of communication that is more concrete and thus may be more readily understood by some children than the use of pictures^36–38^. There is also some evidence that gesture or sign prompts can be effective communication strategies for children with ASC. Most of non-verbal children with ASC would use gestures in the classroom when communicating with teachers^39^. Furthermore, verbal instructions combined with simple gestures and/or signs are reportedly slightly more effective and efficient than verbal instructions alone^25^.

Physical prompts, such as the teacher guiding the hand of a child with their hand, are a feature of the literature in a number of studies promoting learning and communication among children with ASC^40,41^. In such studies, physical prompting is usually associated with earlier more intensive assistance, and thus there is an implicit association of physical prompting with lower levels of independence. Although there are, as far as we can ascertain, no studies which have surveyed the extent to which teachers use physical prompting in autism, nor of their perceptions of its utility, Prizant et al. when discussing the SCERTS model, do note their concerns about the possible negative impacts of teachers using excessive physical prompting^23^. Very few studies have though looked at the use of physical prompting in typical lesson situations where there is free-flow interaction between students and teachers. Chiang, in one of the few instances of such a study, found that physical prompts showed no association with expressive communication from children with ASC, concluding that they may not be a useful instruction^42^. There has been little attention, however, in subsequent studies on empirical evaluation of the role of physical prompts in general teacher-student interactions in autism education.

In this study, we focus on student responsiveness to instruction, i.e., the extent to which a student responds to a teacher’s instruction or other pedagogical techniques, such as modelling how to draw a picture on the whiteboard. This is just one element of communication between teachers and students in the classroom and it may not encompass broader expressive and receptive communication^43^. However, a number of studies have identified responsiveness as a particularly important variable which mediates between classroom instruction and academic outcomes^44–46^. We include a range of attribute data about students, such as sex and age^47^, as well as measures of cognitive and communication development (P-level, SCERTS). P-level is determined by the teachers and is used to assess the language and mathematical ability of children with special educational needs in England^48^. A detailed analysis of P-levels indicates that they are stable in item response theory results across the years^49^. The SCERTS assessment classifies language competence using three stages to represent communication development: (a) social partner—children who engage in non-verbal communication and may communicate intentionally through gestures and vocalisations, (b) language partner—children who communicate with intent using words or word combinations, signs and/or symbols to express meanings, and (c) conversational partner—children who use words, phrases and sentences, and engage in conversations demonstrating an understanding of non-verbal cues of turn taking and topic change^23^. Although SCERTS is based on contemporary practices in autism education, it involves considerable whole-school training at the point of implementation and is supported by high-quality manuals which assist teachers in making judgements on communication stage^50^. As such it has a high degree of ecological validity.

There is also some focus in the literature on how emotion affects student responsiveness^4^. Students with ASC have problems with emotional regulation and may have difficulty controlling their emotional states. This can impact their level of classroom engagement and the extent to which they can successfully respond to classroom instruction^44,51^. Therefore, the student’s observed emotional state is one of the contextual variables considered in the study.

As noted, there is little empirical evidence on how effective different teacher communication strategies are in terms of student responsiveness in autism education. Understanding the extent to which there exist differential levels of responsiveness to various communication strategies may then be helpful in aiding teachers in making decisions about strategy selection which could lead to improved educational outcomes for children with ASC. Our approach not only attempts to provide some insights based on structured observations of teacher-student interactions, but also aims to generalise such findings by developing machine learning solutions that can leverage this information.

## Results

### Task description

Data was collected by observing interactions between teachers and seven students with ASC. To describe an interaction as well as its context, we focused on a specific set of discrete features (categories) and recorded their subtype as listed in Table 1. Categories include student attributes (see Supplementary Table 3), the teaching objective (e.g. academic), the student’s observed emotional state (e.g. negative), the teacher’s communication strategy (e.g. using a picture) or pair of strategies (e.g. verbal and picture), and the student’s response (full, partial or no response). We then used this data to train and evaluate binary classifiers aiming to predict the type of student response based on the rest of the observations. In the binary classification formulation, the two target classes we explored were "full student response” and "either partial or no student response”. Three classifiers were deployed with increasing complexity and expected accuracy capacity: logistic regression with elastic net regularisation (LR), random forest (RF), and a composite Gaussian Process (GP). A detailed description of all the above is provided in the “Methods” section.

**Table 1:** Categories of information that was collected (and subsequently used to build a classifier) by observing interactions between teachers and students with ASC.

Category	Subtypes
Student attributes	Sex, age, P-level, SCERTS
Teaching objective	Academic (*main teaching objective entailed by the observed lesson*), social (*e.g. asking a child to put their shoes on*), pedagogic (*social interaction in pursuit of an academic objective*)
Teaching type	Giving instructions, modelling, redirection, questioning, encouragement/praise, initiating conversation
Context for teaching type	Whole class, small group, individual attention, individual work withdrawal, transition (*e.g. moving to different locations or activities*)
Student’s observed emotional state	Positive (*visibly happy*), negative (*visibly sad*), neutral (*calm with a neutral expression*)
Teacher’s communication strategy	Verbal (*use of words*), gesture (*e.g. pointing with a finger*), physical prompt (*e.g. guiding the arm of a student*), picture (*e.g. using a picture or pointing to a visual chart*), object (*e.g. showing a physical book to encourage the student to get the book*)
Student response (outcome)	Full response (*e.g. when asked to write a sentence in their workbook, the student starts writing*), partial response (*the student starts writing, but then quickly stops*), no response (*the student does* *not engage in writing at all*)

Subtypes (comma-separated) with an explanation in italics (if required) represent the choices or attributes considered for each category.

### Classification accuracy and interpretation

Table 2 enumerates the classification accuracy estimates (10-fold cross-validation) for predicting student response based on the observed data, with (top) and without (bottom) using student attributes (age, sex, P-level, SCERTS), denoted by adding "-*α*” to the end of method abbreviations. The RF classification method delivers the best performance both in terms of raw accuracy and F_1_ score, although there is no statistically significant difference between the RF and the GP outcomes when student attributes are used. In particular, a *t*-test shows that the null hypothesis that RF-*α* is not different from GP-*α* in terms of accuracy and F_1_ score cannot be rejected at *p* = 0.05 (with *p* = 0.847 and *p* = 0.950, respectively). Overall, we see that the classification performance increases for all methods, when student attributes are incorporated. For the RF method, in particular, accuracy and F_1_ score increase by 4.37% and 1.64%, respectively. All outcomes outperform the major class baseline, obtained by classifying everything as "full response”, as the latter delivers an accuracy of 0.566 (SD = 0.023).

**Table 2:** Classification accuracy estimates with their standard deviation (in parentheses) for predicting student response (full response versus otherwise) obtained via a 10-fold cross-validation for the following methods: logistic regression with elastic net regularisation (LR), random forest (RF), Gaussian process (GP), and the same models under an expanded feature set considering student attributes (^⋆^-*α*).

Method	Accuracy	Precision	Recall	F_1_ score
LR	0.647 (0.017)	0.803 (0.013)	0.653 (0.029)	0.720 (0.016)
RF	0.664 (0.017)	0.810 (0.029)	0.669 (0.003)	0.732 (0.012)
GP	0.655 (0.015)	0.804 (0.035)	0.661 (0.023)	0.724 (0.016)
LR-*α*	0.653 (0.014)	0.796 (0.023)	0.661 (0.025)	0.722 (0.012)
RF-*α*	0.693 (0.015)^†^	0.787 (0.025)	0.706 (0.026)	0.744 (0.016)^†^
GP-*α*	0.693 (0.016)^†^	0.787 (0.023)	0.705 (0.027)	0.743 (0.015)^†^

A "†” superscript indicates that there is no statistically significant difference at *p* = 0.05 between estimates (column-wise), after performing a *t*-test.

Table 3 expands on Table 2’s results showing performance estimates when *τ* previous observations and student responses are incorporated. We performed this autoregressive problem formulation for *τ* = {1, …, 5}, and here we present results for the optimal *τ* setting per method (detailed results for all *τ*’s are provided in Supplementary Table 1). All methods are improving compared to their non-autoregressive formulations. We observe a significant performance gain for the GP and GP-*α* methods which outperform the rest, with the exception of the F_1_ score of the GP (*τ* = 1) that does not have a statistically significant difference from the one obtained using the RF (*τ* = 3), with *p* = 0.247. In most occasions, using just a single previous observation provides the most accurate estimates. Notably, the combination of student attributes and autoregression in a GP obtains the best performance in all our experimental setups, yielding an accuracy of 0.711 (SD = 0.015) and an F_1_ score of 0.757 (SD = 0.014). Deploying this best performing model in a leave-one-student-out validation setup (7-fold cross-validation) yields inferior performance as expected given the increased difficulty of the task. However, it still significantly outperforms the major class baseline; more details are provided in the Supplementary Information (SI).

**Table 3:** Classification accuracy estimates with their standard deviation (in parentheses) for predicting student response (full response versus otherwise) incorporating past observations and student responses.

Method	*τ*	Accuracy	Precision	Recall	F_1_ score
LR	1	0.677 (0.017)	0.798 (0.019)	0.684 (0.031)	0.736 (0.015)
RF	3	0.686 (0.014)	0.803 (0.023)	0.692 (0.028)	0.743 (0.014)^†^
GP	1	0.697 (0.015)	0.794 (0.019)	0.708 (0.029)	0.748 (0.013)^†^
LR-*α*	4	0.688 (0.024)	0.790 (0.018)	0.698 (0.032)	0.741 (0.021)
RF-*α*	1	0.701 (0.012)	0.784 (0.028)	0.716 (0.028)	0.748 (0.013)
GP-*α*	1	0.711 (0.015)	0.800 (0.019)	0.720 (0.024)	0.757 (0.014)

The 10-folds are identical to the ones used for obtaining the results presented in Table 2. Results are enumerated for the following methods: logistic regression with elastic net regularisation (LR), random forest (RF), Gaussian Process (GP), and the same models under an expanded feature set considering student attributes (^⋆^−*α*). *τ* denotes the number of previous observations that were used (the best performing models are listed; all results are presented in Supplementary Table 1). A "†” superscript indicates that there is no statistically significant difference at *p* = 0.05 between estimates (column-wise), after performing a *t*-test.

We subsequently perform an ablation analysis of the most accurate classifier (GP-*α*, *τ* = 1) to understand how different feature categories contribute in predicting student response. Its outcomes are enumerated in Table 4. First, we used each category as the only feature for the classifier. Then, we removed one category at a time, computing the classification performance using the remaining ones. In this latter case, the greater the accuracy reduction the more important the feature category is considered to be. In the ablation experiments, we used the same folds as in the original ones, but a simpler formulation of the GP kernel, where a single covariance function is applied to all input data (Eq. (1)). This provides a more straightforward comparison of the impact across different feature categories as it treats the feature space uniformly without segmenting it into subcategories handled by different covariance functions. To this end, we also re-estimated the classification accuracy when a single kernel is applied to all feature categories and used it as an upper-performance benchmark for the ablation analysis (bottom row of Table 4). Past information (observation and student response) is the strongest feature when used in isolation. However, this is most likely due to the fact that it covers the entire feature space, albeit for previous teacher-student interaction instances. Teaching type(s) and the observed emotional state of the student are also good predictors when used alone, in contrast to the teaching objective which yields the worst classification accuracy. When we reverse this experiment, excluding one specific feature category at a time, we can see that omitting the communication strategy of the teacher yields the greatest negative impact to the classifier’s accuracy. The rest of the categories do have a similar impact, with the exception of the teaching objective that by far has the smallest effect. Through a comparison with the suggested upper-performance estimates, we can deduce that all feature categories have a positive contribution to the classifier’s accuracy.

**Table 4:** Ablation analysis for the best performing model GP-*α*, *τ* = 1.

Feature set	Accuracy	Precision	Recall	F_1_ score
Past observations and outcomes	0.631 (0.015)	0.783 (0.022)	0.643 (0.026)	0.706 (0.015)
Teaching type(s)	0.597 (0.015)	0.870 (0.020)	0.599 (0.022)	0.709 (0.015)
Student’s observed emotional state	0.595 (0.023)	0.995 (0.003)	0.583 (0.023)	0.735 (0.018)
Teacher’s communication strategy	0.588 (0.015)	0.784 (0.045)	0.605 (0.026)	0.682 (0.019)
Student’s attributes	0.572 (0.025)	0.883 (0.016)	0.580 (0.027)	0.700 (0.022)
Context	0.572 (0.018)	0.837 (0.021)	0.586 (0.025)	0.689 (0.015)
Teaching objective	0.566 (0.023)	1 (0.000)	0.566 (0.023)	0.722 (0.019)
¬ Teacher’s communication strategy	0.684 (0.022)	0.791 (0.024)	0.694 (0.028)	0.739 (0.017)
¬ Context	0.693 (0.012)	0.785 (0.020)	0.707 (0.030)	0.743 (0.010)
¬ Past observations and outcomes	0.693 (0.015)	0.790 (0.020)	0.704 (0.026)	0.744 (0.014)
¬ Student’s attributes	0.695 (0.018)	0.795 (0.016)	0.705 (0.034)	0.747 (0.014)
¬ Student’s observed emotional state	0.696 (0.016)	0.784 (0.019)	0.709 (0.030)	0.744 (0.017)
¬ Teaching type(s)	0.696 (0.019)	0.802 (0.022)	0.704 (0.030)	0.749 (0.015)
¬ Teaching objective	0.705 (0.018)	0.794 (0.019)	0.716 (0.030)	0.752 (0.018)
All features	0.707 (0.015)	0.796 (0.020)	0.718 (0.026)	0.754 (0.014)

A single kernel function is used (Eq. (1)) across the entire feature space to allow a straightforward interpretation of the results. The upper part of the Table enumerates accuracy estimates when a single feature category is used. At the lower part, "¬” denotes the absence of the specified feature category. For all estimates we have also included their standard deviation (in parentheses).

### Long-term teacher communication strategy effect via a statistical analysis

**Fig. 1:**
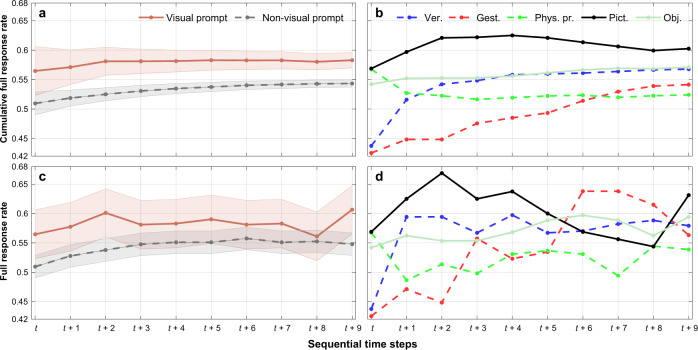
Long-term effects of teacher communication strategies. **a** Cumulative student full response rate with 95% confidence intervals (shaded) across a sequence of consecutive teacher communications, where the first one is a visual prompt (solid line) or a non-visual prompt communication strategy (dashed line); **b** Cumulative student full response rate across a sequence of consecutive teacher communications, for all possible first teacher communication strategy options; **c** Student full response rate with 95% confidence intervals (shaded) for all points within a sequence of consecutive teacher communications, where the first one is a visual prompt or a non visual prompt communication strategy; **d** Student full response rate for all points within a sequence of consecutive teacher communications, for all possible first teacher communication strategy options. For both (**a**) and (**b**), the averaging is progressive as new data points become available (cumulative) and thus, it is expected to converge to the actual average full response rate in the data (56.59%). In contrast, for both (**c**) and (**d**), the averaging is focused on each single sequence point and thus, it is not expected to converge to a certain quantity.

The immediate effect of a communication strategy can be misleading as in the relative long-term it may be inducing no or a negative impact. To better understand this, for any type of teacher communication engaged at a specific time step *t*, we computed the cumulative full student response rate for up to 9 consecutive future time steps; including *t*, these are time steps {*t*, *t* + 1, …, *t* + 9}. By cumulative, we refer to the ratio of full responses in an additive fashion across a sequence of these 10 time steps. Note that the initial communication belongs to a certain category only, i.e., instances with secondary actions are not considered as eligible starting points; during the following time steps (*t* + 1 to *t* + 9), any other communication strategy (single or a pair) may be performed. As this is a cumulative quantity, we expect it to eventually converge to the average full response rate in the data (56.59%). Apart from looking at each teacher’s communication separately, we also grouped communication strategies to visual prompts (object, picture) and non-visual prompts. This grouping was motivated by two observations: (a) full student response rates in our data were at their highest level when visual prompts were applied, and (b) the conditional distributions of the probability of full student response given either of the two visual prompts (picture, object) were very similar (see also our statistical analysis and Fig. 3c). For completeness, we repeated this analysis for step-wise, non-cumulative full student response rates, which are not expected to converge to a certain quantity, but at the same time are providing more noisy information. Figure 1 presents the outcome of this analysis. Overall, visual prompts appear as more effective in generating full student responses at the time of their application (*t*) as well as at later time steps. Interestingly, visual prompts are enhancing their positive impact one and two time-steps (*t* + 1, *t* + 2) after their original application (Fig. 1a, c). Non-visual prompts show a more spread incline, up to *t* + 4. By looking at individual communications (Fig. 1b, d), we see that the use of pictures has been the most effective. Furthermore, sequences that begin with a physical prompt, although they initiate strong student response rates at the time of their application, as the sequence of communications progresses, always end up with the lowest rates either cumulatively (from time step *t* + 7) or otherwise (from time step *t* + 6).

We also assessed whether the previously presented outcomes could be due to the hierarchies teachers might deploy in their communication strategies. For example, when a non-intrusive prompt (e.g. a verbal communication) does not produce the desired outcome, it could be postulated that they might attempt to use a more intrusive one (e.g. a physical prompt). Then, when an intrusive prompt succeeds, they may revert back to using less intrusive prompts. This could justify why in the second time step (*t* + 1) we observe a steep increase in full student response for non-intrusive communications on the lower end (e.g. verbal), and a noticeable decrease for physical prompts (Fig. 1b, d). However, our data do not fully support this hypothesis. In particular, when a teacher communication, other than a physical prompt, does not result in a full student response, then the subsequent communication contains a physical prompt only with a probability of 0.310 and when that happens the probability of a full student response is 0.549 (success rate). In general, when a teacher’s communication is not successful, then the subsequent communication contains a verbal, visual, physical, and gesture prompt with respective probabilities of 0.579, 0.405, 0.370, and 0.297 (with 0.374, 0.453, 0.496, and 0.415 respective success rates). Hence, physical prompts are not necessarily the most common choice when students are not responsive. Notably though, when a communication is successful, physical prompts are very rarely utilised subsequently (0.188). Additional statistics for sequential patterns of communications in our data are provided in the SI.

### Using student response to recommend teacher communication strategies

**Fig. 2:**
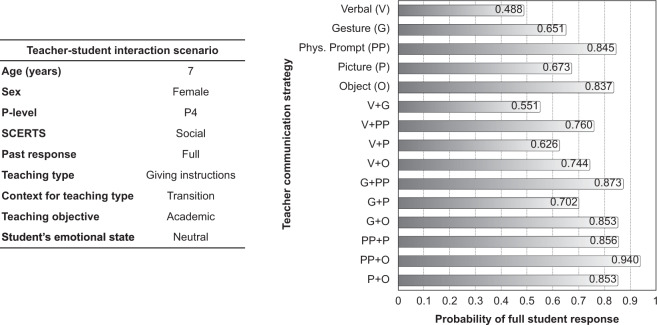
An example of using the machine learning classifier for predicting the non-calibrated probability that one or more teacher communication strategies would result in a full student response. The scenario parameters are listed in the table on the left—note that the student profile and the characteristics of this teacher-student interaction are both out-of-sample (i.e. not existent in the collected data). The chart on the right depicts the probabilities of full student response (for the interaction described on the left) for all single teacher communication strategies as well as all possible pairings of them.

The GP classifier outputs a real number that ranges from [−1, 1] and indicates the specific support for a classification outcome. For example, a value equal to 0.5, which results to a full student response classification as it is >0, can be mapped to a 0.75 probability for this outcome. We acknowledge that this might result into biased face values for these conditional probabilities^52^, but our approach utilises their relative ranking during the decision-making process which remains unaffected. For a given teacher-student interaction instance, we can change the input of the classifier such that all the different communication strategies or pairs of them are activated one at a time, and obtain a set of full student response probabilities each conditioned on the selected communication(s). We can then choose the one that has the greatest probability of generating a full student response. That way our model can produce teacher communication strategy suggestions for specific teacher-student interaction cases. Figure 2 provides an example to showcase this. The teacher-student interaction scenario is listed on the left, and the corresponding probability of full student response given each possible teacher communication or pair of communications, $\Pr \left(\,{{\mbox{full response}}}| {{\mbox{teacher communication(s)}}}\,\right)$, is presented on the right. These estimates were based on the best-performing model (GP-*α*, *τ* = 1), trained on all the collected data. This particular scenario under investigation was not present in the collected data, i.e., it is a simulated observation (more examples are provided in Supplementary Table 2). In this example, the next best communications to a physical prompt (that might not be a desirable communication strategy) with quite strong probabilities of success are "object” when one teacher communication is performed (Pr = 0.837), or one of the combinations of "gesture and object” or "picture and object” (Pr = 0.853 for both) when two communications are performed.

### Statistical insights through the lens of the classifier

**Fig. 3:**
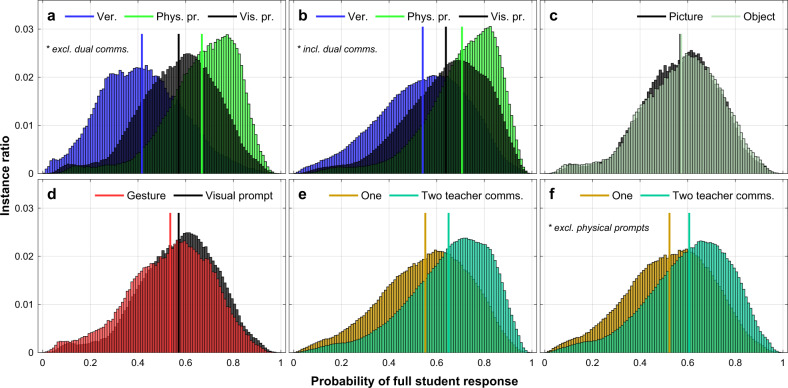
Probability distribution of full student response under various scenarios, using the machine learning classifier. The following scenarios are explored: **a** comparison between verbal teacher communications, physical, and visual prompts when only a single communication is applied; **b** comparison between verbal teacher communications, physical, and visual prompts, including instances where two (dual) communications are applied; **c** comparison between picture and object teacher communications (i.e. between the two visual prompts); **d** comparison between gestures and visual prompts; **e** comparison between one and two teacher communications; **f** comparison between one and two teacher communications when communications with a physical prompt are excluded. Straight vertical lines denote the mean of the corresponding distribution.

The collected data may include a biased representation of teacher-student interaction instances and certainly cannot explore all possible scenarios and teacher communication strategies. Using our task formulation (as shown in Table 1), there exist 90720 different interaction scenarios, which when coupled with all possible teacher communications in our problem formulation (a total of 15 when dual communications are considered) are generating more than 1.3 million distinct observations. However, our collected data cover only 4880 of these (see “Methods”). The number of possible observations increases further as we begin to include past observations. To make a more robust analysis, unveiling trends that might have been suppressed in the collected data, we sampled this large feature space, and generated a representative amount of more than 2.6 million unique observations. We used the best-performing model (GP-*α*, *τ* = 1) trained on all the collected instances to determine the student response for each one of these sampled observations. In this expanded data set, the features with the greatest absolute correlation with student response were the negative student emotion state (*r* = −0.431, *p* ≪ 0.001), the physical prompt communication (*r* = 0.341, *p* ≪ 0.001), the verbal communication (*r* = −0.292, *p* ≪ 0.001), and the encouragement/praise teaching type (*r* = 0.225, *p* ≪ 0.001). In addition to correlations that were already present in the original data set (see “Methods”), the machine learning method also picked up two patterns related to specific teacher communication strategies. The verbal communication was anti-correlated with full student response contrary to the physical prompt communication that had the greatest positive correlation. Figure 3 depicts the probability distribution of full student responses for different teacher communications. We can see that physical prompts are the most effective either as a single communication or in combination with others (Fig. 3a, b). However, this is expected given that this is the most intrusive prompt. Verbal communications are the least effective. Visual prompts are in a median position, although closer to the efficiency of physical prompts, especially when dual communications are considered. Interestingly, the two visual prompts (picture, object) have almost identical full student response probability distributions (Fig. 3c). This encourages their consideration as one communication category (visual prompts), but also highlights that the classifier has picked up very similar patterns in the generalised conditional distributions, Pr(full student response∣picture) and Pr(full student response∣object). Visual prompts are also more effective compared to gestures, but the discrepancy is relatively small (Fig. 3d). Finally, two teacher communications are a better option than one, with (Fig. 3e) or without (Fig. 3f) considering physical prompts.

## Discussion

A machine learning classifier was able to predict which type of teacher communication was more likely to generate a positive response by a student with ASC, indicating that the student responded to the communication in a way intended by the teacher, with an accuracy (0.664; RF model) greater than that expected from a random (0.500) or major class (0.566) baseline prediction. When student attributes, i.e., cognitive and language levels, sex and age, are added into the function, the accuracy level increases (0.693; RF or GP model), and when past information is incorporated, accuracy improves further (0.711; GP-*α*, *τ* = 1). Thus, the results of this exploratory research indicate that the developed classifier, derived from observations of teacher-child interactions, has the capacity to capture relevant signals from the data, which is instrumental for its potential usefulness in classroom practice. Based on the ablation analysis, teacher communications did indeed have the greatest impact on classification accuracy (3.25% of reduction on average), something the reinforces the importance of choosing the right type of communication.

The statistical analysis of consecutive observations (teacher communication type → student response) indicated that for visual prompts (using a picture or an object) the observed consecutive full response rate increases in the short-term, and converges to a maximum (compared to all other communications) two-time steps after its application. In contrast, physical prompts can have an immediate positive impact followed by a significant performance drop thereafter. In addition, for longer sequences that begin with a physical prompt, the return to the mean is at a lower level of full response, when compared to other teacher communication strategies. Thus, the data suggests that the use of physical prompts leads to less engagement by children with ASC, both at the initial use of the prompt, and for subsequent interaction, at least within the limits of the sequence size considered here. As noted, there is general support in the literature for the potential efficacy of visual aids in developing communication and autism education, as well as implicit concerns about the potential impact of physical prompting on independence. The results presented here, however, provide empirical support for the extent to which different teacher communication strategies in general classroom situations have an impact on student responsiveness.

Our data also indicates that a full student response is more likely with two rather than one teacher communication. This aligns with related studies suggesting that verbal instruction by itself is less effective with children with ASC^25,26^. In addition, it coincides with the "common-sense” perspective that was pointed out by the teachers involved in the study, i.e., that they would typically expect to see better responsiveness when two communications are used. The fact that the machine learning model produces outcomes that coincide with what was generally expected serves as further evidence to support its ecological validity.

The key potential strength of this model is in the possibility for teachers, in advance of a teaching session, to input specific variables, as in Fig. 2, relating to real-world scenarios in the classroom, for a specific child with specific attributes and conditions, and then review the recommendations of the model for specific teacher communications. If this exercise provides information that helps the teachers make more effective decisions which, in turn, promote the effective inclusion of children with ASC, then such an approach could make a real difference in autism education. Our study has shown that there is potential for the use of a machine learning model in this way.

There are few empirical studies that compare the relative effectiveness of multiple different teacher communication strategies in autism education, with most studies focusing on one or two individual strategies. Our study is innovative in its use of a machine learning approach to undertake a comparison of multiple strategies. Many, if not most, studies in the field focus on the use of specific interventions which involve the implementation of a set of specified steps, often delivered in a discrete setting, i.e., in a separate room next to the classroom with a teaching assistant, and for a specific time period^25,40,53^. However, the vast majority of the teaching of children with ASC takes place in general classroom situations, not in relation to specific interventions^54^. Hence, our focus on the use of different strategies in the free flow of classroom interaction can shed further light on effective practice.

To the best of our knowledge, this is the first time that data from direct observation of children with ASC and their teachers in the classroom have been used in the development of a machine learning model. To this end, our study is exploratory and has specific limitations. Primarily, the results are based on a cohort of seven students which is a relatively small number, and thus any claims of generalisation need to be approached with caution. We attempted to mitigate the effects of this, to an extent, by collecting a significant amount of observations (>5000). In addition, the attributes used to represent students are not standardised metrics based on a clinical diagnosis, but instead are educational indicators commonly used in UK schools, although as argued they do have a degree of ecological validity. There may well be other categories that could potentially be included in the observation schedule such as the proximity between child and teacher (via video analysis) or known child preferences such as sensitivity to noise or light. It is, of course, the case, that expanding the number of dependent categories in the model or its overall complexity is likely to increase accuracy, but the corollary to this is the requirement to collect and code a significantly increased amount of observations. Clearly, larger cohorts that encompassed a wider range of phenotypical variability in the expression of ASC would allow for a more fine-grained and robust analysis of the influence of student attributes, and particularly of their developmental profile, on the accuracy of the function’s recommendations. It will also enable a more concise analysis of the longer-term effects of teacher communications.

## Methods

### Data collection

A data set was formed through structured classroom observations in 20 full-day sessions over 5 months in 2019 at a special school with criteria of ASC for admission in East London. Participants included three teachers (one male, two females), their teaching assistants (all females), and seven children (four males, three females) aged from 6 to 12 years across 3 classes. The children’s P-scales range from P3 to P6; P-scale commonly ranges from P1 to P8, with P1–P3 being developmental non-subject-specific levels, and with P4–P8 corresponding to expected levels for typical development at ages 5–6^48^. In addition, the children are also described as social or language partners on the SCERTS scale used by the school. In our study, none of the participating students were classified as conversational partners. The attributes of the student cohort are presented in Supplementary Table 3.

A coding protocol was developed through an iterative process with the participating teachers, and a grid was used for recording teacher-student interaction observations. Comments and suggestions from the teachers were taken into consideration and reflected throughout the multiple revised drafts and the final versions of the coding protocol and recording grid. For each observation instance, we recorded the student identifier, time stamp, teaching objective, teaching type, the context for this teaching type, the student’s observed emotional state, teacher’s communication strategy, and the corresponding student response (outcome). Where applicable we also recorded additional notes and the type of activity (e.g. yoga). Although notes were used for context and interpretation for the data analysis as a whole, they were not included in our machine learning function experiments given their free-form inconsistency. Table 1 details all the subcategories that were considered as inputs to the machine learning models. Up to two teaching types and teacher communications could be attributed to a single observation; the rest of the categories can only be represented by one subtype. For example, an observation coded as "3, academic, giving instruction/modelling, whole class, positive, verbal/gesture, full response” (the time stamp is omitted) represents that student no. "3”, being in a positive emotional state, fully responded to a teacher’s verbal and gesture instruction, when teaching was taking place in a whole class environment, its type was modelling and had an overall academic objective. This may refer to an interaction instance where the teacher is delivering a yoga lesson to the whole class: the teacher is demonstrating a yoga move by gesturing while verbally explaining it and asking the students to do the same; the student then responds by doing the move with an observably happy expression.

All observed adult-student interactions during the school day, permitted by the teachers, were recorded. The aim was to rapidly record situation-strategy-outcome data points "in vivo” inside and outside the classroom. Locations of the observations outside the classroom include the playground, library, music room, main hall, canteen, therapy rooms, and garden. Overall, these resources were regularly used throughout the observational sessions. The instances recorded for each student vary slightly from 753 to 880 (*μ* = 780, *σ* = 45) and in total a sample of 5460 full observations were collected.

### Statistical characterisation of collected data

From the 5460 observations we collected, only 5001 are distinct. If we ignore the student’s response, unique observations are reduced to 4880, and if we also ignore the teacher’s communication strategy, then this number becomes 4357. Hence, there are instances in our data that are overlapping, but this is expected given that teachers and students may perform similarly throughout a specific teaching session. The level of support for each teacher communication strategy is equal to 3128 (709) times for a verbal communication, 1717 (357) for using an object, 1642 (181) for the gesture, 1465 (575) for a physical prompt, and 981 (165) for a picture, where in parentheses we report the number of times the underpinned communication was the only one performed (from a maximum of two communications). Although the small student and teacher sample does not allow for generalisations, we see that teachers tend to verbally engage with students quite frequently (57.29%), either in combination with another communication or as the sole means of communication. The full student response rate for each communication strategy (irrespectively of co-occurrence with another one) is equal to 64.02% (64.90%, 60.68%) for picture, 60.92% (62.48%, 57.73%) for an object, 60.61% (64.34%, 53.56%) for a physical prompt, 57.67% (59.67%, 51.80%) for a gesture, and 53.20% (55.21%, 46.45%) for a verbal communication; the rates in the parentheses are breakdowns for the language and social partner SCERTS classifications, respectively, reaffirming those language partners are in general more responsive, with a more pronounced relative difference when verbal or physical prompts are deployed. In addition, performing two versus one communication is more effective in producing a full student response. In particular, the full, partial, and no response breakdowns for single communications are 50.58%, 21.84%, and 27.58%, compared to 60.01%, 21.82%, and 18.17% for two teacher communications. Although the presence of two communications naturally increases the probability of choosing the correct means of interaction, the current outcome reaffirms the hypothesis that an incorrect communication strategy does not greatly affect the student when a desirable one co-occurs. The observed features with the greatest bivariate correlation with the student response are the negative emotional state of the student (*r* = −0.184, *p* ≪ 0.001), the encouragement/praise teaching type (*r* = 0.124, *p* ≪ 0.001), and the redirection teaching type (*r* = −0.124, *p* ≪ 0.001).

### Student response (outcome) classification with machine learning

A machine learning classification task aims to learn a function *f*: **X** → **y**, where ${{{\bf{X}}}}\in {{\mathbb{R}}}^{m\times n}$, **y** ∈ {1, …, *k*}^*m*^ denote the observations (inputs) and the response variable (outcomes), respectively; *m*, *n*, *k* represent the numbers of observations and outcomes, observation categories (features), and outcome classes, respectively. Here, in the most feature-inclusive case, we define **X** as an aggregation of six feature categories, namely student attributes (age, sex, P-level, SCERTS classification), teaching objective, teaching type, context for teaching type, the student’s observed emotional state, and teacher’s communication strategy. All feature categories, apart from age, were coded as *c*-dimensional tuples of 1s and 0s, where *c* is the respective number of different subtypes for each category (Table 1), and ones are used to denote the activated subtype(s). Student age was coded as a real number from 0 to 1, using a linear mapping scheme, where 0 and 1 represent 5 and 12 years of age, respectively. The response variable **y** takes a binary definition representing two classes, a full response output versus otherwise. The rational behind this merging was to generate a more balanced classification task (56.59% full student response labels) as well as alleviate any issues arising from a miscategorisation of partial (21.86%) or no response (21.55%) outcomes.

We train and evaluate the performance of various machine learning functions in predicting the student’s type of response. We deploy three broadly used classifiers in the literature: (a) a variant of logistic regression (LR)^55^ that uses elastic net regularisation^56^ for feature selection, (b) a random forest (RF)^57^ with 2000 decision trees, and (c) a Gaussian Process (GP)^58^ with a composite covariance function (or kernel) that we describe below. We devise three problem formulations, where we incrementally add more elements in the observed data (input). In the first instance, we consider all observed categories apart from student attributes. Then, we include student attributes as part of the feature space and, to represent this change, augment method abbreviations with "-*α*”. Finally, in both previous setups, we explore autoregression by including the observed data and student responses for up to the previous *τ* = 5 teacher-student interactions. While performing autoregression, we maintain all three types of recorded student responses in the input data.

Although logistic regression and random forests treat the increased input space without any particular intrinsic additive modelling, the modularity of the GP allows us to specify more customised covariance functions on these different inputs. GP models assume that *f*: **X** → **y** is a probability distribution over functions denoted as $f({{{\bf{x}}}}) \sim \,{{\mbox{GP}}}\,(\mu ({{{\bf{x}}}}),k({{{\bf{x}}}},{{{\bf{x}}}}^{\prime} ))$, where ${{{\bf{x}}}},{{{\bf{x}}}}^{\prime}$ are rows of **X**, *μ*(⋅) is the mean function of the process, and *k*(⋅,⋅) is the covariance function (or kernel) that captures statistical relationships in the input space. We assume that *μ*(**x**) = 0, a common setting for various downstream applications^59–62^, and use the following incremental (through summation) covariance functions:

1 $$k({{{\bf{x}}}},{{{\bf{x}}}}^{\prime} )={k}_{{{{\rm{SE}}}}}({{{{\bf{x}}}}}_{c},{{{{\bf{x}}}}}_{c}^{\prime})\ ,$$

2 $$k({{{\bf{x}}}},{{{\bf{x}}}}^{\prime} )={k}_{{{{\rm{SE}}}}}({{{\bf{a}}}},{{{\bf{a}}}}^{\prime} )+{k}_{{{{\rm{SE}}}}}({{{{\bf{x}}}}}_{c},{{{{\bf{x}}}}}_{c}^{\prime})\ ,$$

3 $$k({{{\bf{x}}}},{{{\bf{x}}}}^{\prime} )={k}_{{{{\rm{SE}}}}}({{{{\bf{x}}}}}_{c},{{{{\bf{x}}}}}_{c}^{\prime})+{k}_{{{{\rm{SE}}}}}({{{{\bf{x}}}}}_{p},{{{{\bf{x}}}}}_{p}^{\prime})+{k}_{{{{\rm{SE}}}}}({{{{\bf{y}}}}}_{p},{{{{\bf{y}}}}}_{p}^{\prime})\ ,\,{{\mbox{and}}}\,$$

4 $$k({{{\bf{x}}}},{{{\bf{x}}}}^{\prime} )={k}_{{{{\rm{SE}}}}}({{{\bf{a}}}},{{{\bf{a}}}}^{\prime} )+{k}_{{{{\rm{SE}}}}}({{{{\bf{x}}}}}_{c},{{{{\bf{x}}}}}_{c}^{\prime})+{k}_{{{{\rm{SE}}}}}({{{{\bf{x}}}}}_{p},{{{{\bf{x}}}}}_{p}^{\prime})+{k}_{{{{\rm{SE}}}}}({{{{\bf{y}}}}}_{p},{{{{\bf{y}}}}}_{p}^{\prime})\ ,$$

where *k*_SE_(⋅,⋅) denotes the squared exponential covariance function, **x**_*c*_ denotes the current observation including the teacher’s communication strategy, **a** is the vector containing student attributes, and **x**_*p*_, **y**_*p*_ denote the *τ* past observations and student response outcomes, respectively. Therefore, Eq. (1) refers to the kernel in the simplest task formulation where only currently observed data are used, Eq. (2) expands on Eq. (1) by adding a kernel for student attributes, and Eqs. (3) and (4) add kernels for including previous observations and student responses (autoregression). Using an additive problem formulation, where a kernel focuses on a part of the feature space, generates a simpler optimisation task and tends to provide better accuracy^63^. This is also confirmed by our empirical results.

### Training and evaluating classifiers

We apply 10-fold cross-validation as follows. We randomly shuffle the observed samples (5460 in total) and then generate 10 equally sized folds. We use 9 of these folds to train a model, and 1 to test, repeating this training-testing process 10 times, using all formed folds as test sets. By doing this we are solving a task, whereby observations from the same student can exist in both the training and the test sets (although these observations are strictly distinct). That was an essential compromise here given the limited number of different students (7). The same exact training and testing process (and identical data splits) is used for all classification models and problem formulations. We learn the regularisation hyperparameters of logistic regression by cross-validating on the training data; this may result in potentially different choices for each fold. The hyperparameters of the GP models are learned using the Laplace approximation^58,64^. Performance is assessed using standard classification metrics, and in particular accuracy, precision, recall, and their harmonic mean known as the F_1_ score. For completeness, we also assess the best-performing model by testing on data from a single student that is not included in the training set, repeating the same process for all students in our cohort (leave-one-student-out, 7-fold cross-validation; see SI for more details).

### Ethics approval

Ethical approval was granted by the Research Ethics Committee at the Institute of Education, University College London (United Kingdom), where the research was conducted. The parents/guardians of the participating children, the school management, and their teachers gave their written informed consent. All participant information has been anonymised. Raw data and derived data sets were securely stored on the researchers’ encrypted computer systems with password protection.

### Reporting summary

Further information on research design is available in the Nature Research Reporting Summary linked to this article.

## Supplementary information

Supplementary Information

Reporting Summary

## Data Availability

The data sets generated during and/or analysed during the current study are not publicly available due to their sensitive nature and cannot be shared upon request as this would require specific new written consent from the parents of each child.

## Code availability

We have used standard and contributed MATLAB^®^ (R2019a) functions or libraries to conduct experiments. In particular, we have used function lassoglm for logistic regression with elastic net regularisation, function TreeBagger for the random forest, and the Gaussian Process Regression and Classification Toolbox (version 4.2, gaussianprocess.org/gpml/code/matlab/doc/) for deriving GP models.
