# Supplementary material for: An artificial intelligence approach for selecting effective teacher communication strategies in autism education
Source: NPJ Sci Learn. 2021 Sep 1;6:25. doi: 10.1038/s41539-021-00102-x (PMC8410830; doi:10.1038/s41539-021-00102-x)
Supplement: Supplementary file 1 — Supplementary Information [file 41539_2021_102_MOESM1_ESM.pdf]

Supplementary Information for “*An artificial intelligence approach for selecting effective teacher communication strategies in autism education*”

Vasileios Lampos[1,*,†], Joseph Mintz[2,*,†], and Xiao Qu[2]

[1]Department of Computer Science, University College London, London, UK
[2]Institute of Education, University College London, London, UK
[†]These authors contributed equally
[*]Corresponding authors: v.lampos@ucl.ac.uk, j.mintz@ucl.ac.uk

Supplementary Methods

Additional evaluation of the student response classification task

For completeness, we have performed an experiment where we used the best performing model in our current validation setup (GP-α, $\tau = 1$, i.e. the Gaussian Process that incorporates student attributes and one previous observation), trained strictly on data from 6 students, and tested on data from the remaining 1 student. We repeated this process 7 times, testing on a different student each time, and training with the remaining data instances (7-fold cross validation). We obtained a mean accuracy of .665 (SD $=$ .043), a precision of .765 (SD $=$ .073), a recall of .687 (SD $=$ .095), and an F_1-score of .717 (SD $=$ .043). The corresponding major class baseline is equal to .563 (SD $=$ .072) and this model clearly outperforms it.

Additional sequential patterns of teacher communications in relation to student response

Some additional observations that supplement the Results subsection “Long-term teacher communication strategy effect via a statistical analysis” are provided below. When a teacher communication results in a full student response, then the subsequent communication contains:

- a verbal prompt with a probability of .593 and when that happens the probability of a full student response (success rate) is equal to .646,
- a visual prompt with a probability of .550, and a success rate of .714,
- a gesture with a probability of .304, and a success rate of .697, and
- a physical prompt with a probability of .188, and a success rate of .777.

When visual prompts fail to produce a full student response, the subsequent action contains:

- a physical prompt, but not a visual prompt, with a probability of .212 and a success rate of .490, and
- a visual prompt, but not a physical prompt, with a probability of .481 and a success rate of .405.

The above indicates that an unsuccessful visual prompt is more likely to be followed by another visual prompt than a physical prompt. Finally, when a physical prompt is successful, then the subsequent communication when considering only single communications is:

- a visual prompt with a probability of .483 and a success rate of .503,
- a verbal communication with a probability of .407 and a success rate of .566,
- a physical prompt with a probability of .334 and a success rate of .679, and
- a gesture with a probability of .309 and a success rate of .571.

Supplementary Tables

Supplementary Table 1 is an expanded version of Table 3 of the main manuscript. Here we provide performance estimates for all values of τ that we considered, i.e. $\tau \in \{1, \ldots, 5\}$; τ denotes the number of past observations that were used as additional inputs to the student response classifier.

Supplementary Table 2 is an expanded version of Figure 2 of the main manuscript, providing 3 additional teacher-student interaction scenarios of method deployment (4 in total). The teacher-student interaction scenarios —generated such that they were not present in the collected observations— are listed in the top table, and the corresponding non-calibrated probability of full student response given each possible teacher communication or pair of communications, $\Pr(\text{full response}|\text{teacher communication(s)})$, at the bottom table. These estimates were based on the best performing model (GP-α, $\tau = 1$), trained on all the collected data. Given that a physical prompt might not always be a desirable communication strategy, we have also underlined different options when physical prompts received the highest probability score for a full student response. According to the classifier, Cases A and D are easier to resolve as most communication strategies will yield a high probability of full student response ($\Pr(\cdot) > .5$). Case C requires a combination of communications, whereas for Case B, where the student's emotional state is negative, all teacher communications seem to be less effective ($\Pr(\cdot) < .5$).

Supplementary Table 3 lists the attributes of the students that participated in our study, showcasing that our cohort has been relatively heterogeneous.

Method	τ	Accuracy	Precision	Recall	F_1 score
LR	1	.677 (.017)	.798 (.019)	.684 (.031)	.736 (.015)
	2	.676 (.014)	.792 (.024)	.685 (.025)	.734 (.015)
	3	.675 (.018)	.791 (.026)	.684 (.026)	.733 (.016)
	4	.676 (.021)	.793 (.027)	.685 (.029)	.734 (.019)
	5	.675 (.022)	.793 (.024)	.684 (.030)	.734 (.019)
RF	1	.684 (.019)	.769 (.027)	.702 (.035)	.733 (.015)
	2	.684 (.014)	.787 (.023)	.696 (.028)	.738 (.011)
	3	.686 (.014)	.803 (.023)	.692 (.028)	.743 (.014)†
	4	.682 (.018)	.813 (.019)	.685 (.029)	.743 (.017)
	5	.681 (.022)	.819 (.016)	.682 (.032)	.744 (.020)
GP	1	.697 (.015)	.794 (.019)	.708 (.029)	.748 (.013)†
	2	.691 (.014)	.789 (.021)	.703 (.027)	.743 (.012)
	3	.688 (.010)	.789 (.019)	.699 (.023)	.741 (.008)
	4	.692 (.015)	.793 (.020)	.702 (.027)	.744 (.013)
	5	.686 (.017)	.789 (.018)	.697 (.028)	.740 (.014)
LR-α	1	.686 (.018)	.793 (.021)	.696 (.030)	.741 (.017)
	2	.682 (.018)	.791 (.022)	.692 (.030)	.738 (.017)
	3	.685 (.020)	.792 (.019)	.695 (.032)	.740 (.018)
	4	.688 (.024)	.790 (.018)	.698 (.032)	.741 (.021)
	5	.682 (.022)	.787 (.017)	.693 (.029)	.737 (.020)
RF-α	1	.701 (.012)	.784 (.028)	.716 (.028)	.748 (.013)
	2	.698 (.020)	.794 (.023)	.708 (.028)	.748 (.017)
	3	.700 (.016)	.814 (.021)	.703 (.029)	.754 (.015)
	4	.696 (.017)	.817 (.021)	.698 (.029)	.752 (.015)
	5	.690 (.017)	.824 (.020)	.689 (.029)	.750 (.017)
GP-α	1	.711 (.015)	.800 (.019)	.720 (.024)	.757 (.014)
	2	.704 (.015)	.792 (.017)	.716 (.026)	.751 (.014)
	3	.699 (.015)	.794 (.016)	.709 (.027)	.749 (.013)
	4	.696 (.015)	.791 (.016)	.707 (.027)	.746 (.013)
	5	.695 (.014)	.791 (.015)	.707 (.028)	.746 (.012)

Supplementary Table 1. Classification accuracy estimates with their standard deviation (in parentheses) for predicting student response (full response *versus* otherwise) incorporating past observations and student responses. Results are enumerated for the following methods: logistic regression with elastic net regularisation (LR), random forest (RF), Gaussian Process (GP), and the same models under an expanded feature set considering student attributes ($\star$-α). τ denotes the number of previous observations that were used. A "†" superscript indicates that there is no statistically significant difference at $p = .05$ between estimates (column-wise), after performing a t-test.

**Attributes / Examples**	**Case A**	**Case B**	**Case C**	**Case D**
Age (years)	7	6	9	10
Sex	Female	Female	Male	Male
P-level	P4	P3	P3	P5
SCERTS	Social	Language	Social	Language
Past response	Full	Full	Partial	No response
Teaching type	Giving instructions	Modelling	Redirection	Modelling
Context for teaching type	Transition	Ind. attention	Ind. attention	Ind. attention
Teaching objective	Academic	Academic	Social	Pedagogic
Student’s emotional state	Neutral	Negative	Neutral	Positive

**Teacher communication strategy**					**Pr(full response | teacher communication(s))**			
**Verbal**	**Gesture**	**Phys. prompt**	**Picture**	**Object**	**Case A**	**Case B**	**Case C**	**Case D**
✓	–	–	–	–	.488	.252	.259	.618
–	✓	–	–	–	.651	.272	**.409**	.728
–	–	✓	–	–	**.845**	.356	.399	.719
–	–	–	✓	–	.673	**.361**	.303	.708
–	–	–	–	✓	.837	.299	.375	**.744**
✓	✓	–	–	–	.551	.299	.459	.751
✓	–	✓	–	–	.760	.364	.429	.711
✓	–	–	✓	–	.626	.394	.358	.738
✓	–	–	–	✓	.744	.311	.449	.762
–	✓	✓	–	–	.873	.405	**.592**	.828
–	✓	–	✓	–	.702	.396	.484	.810
–	✓	–	–	✓	.853	.345	.558	**.845**
–	–	✓	✓	–	.856	**.458**	.454	.781
–	–	✓	–	✓	**.940**	.424	.514	.813
–	–	–	✓	✓	.853	.423	.440	.806

Supplementary Table 2. Four examples (Cases A-D) of using the machine learning classifier for predicting the non-calibrated probability that one or more teacher communication(s) would result to a full student response. The settings of each case are described on the top table – note that the attributes of these students are not exact matches of the ones in our cohort. The bottom table enumerates the probabilities of full student response for all single communications as well as all possible pairings of them. Bold font indicates the communication or pair of communications with the greatest probability for yielding a full student response. Underlined outcomes are showing the same, when the physical prompt communication is ignored.

Sex	Age	SCERTS	P-level
Female	11 years, 7 months	Social	P3
Female	11 years, 4 months	Language	P6
Male	11 years	Language	P6
Male	9 years, 6 months	Social	P5
Male	9 years, 1 month	Language	P6
Male	7 years	Language	P6
Female	5 years, 8 months	Social	P4

Supplementary Table 3. The study's participants and their attributes. To calculate age we used a reference month in 2019.
