# Supplementary material for: An artificial intelligence approach for selecting effective teacher communication strategies in autism education
Source: NPJ Sci Learn. 2021 Sep 1;6:25. doi: 10.1038/s41539-021-00102-x (PMC8410830; doi:10.1038/s41539-021-00102-x)
Supplement: Supplementary file 2 — Reporting Summary [file 41539_2021_102_MOESM2_ESM.pdf]

nature research

Corresponding author(s): Vasileios Lampos
Joseph Mintz

Last updated by author(s): Apr 27, 2021

Reporting Summary

Nature Research wishes to improve the reproducibility of the work that we publish. This form provides structure for consistency and transparency in reporting. For further information on Nature Research policies, see our Editorial Policies and the Editorial Policy Checklist.

Statistics

For all statistical analyses, confirm that the following items are present in the figure legend, table legend, main text, or Methods section.

n/a	Confirmed	
☐	☒	The exact sample size (n) for each experimental group/condition, given as a discrete number and unit of measurement
☐	☒	A statement on whether measurements were taken from distinct samples or whether the same sample was measured repeatedly
☐	☒	The statistical test(s) used AND whether they are one- or two-sided<br>*Only common tests should be described solely by name; describe more complex techniques in the Methods section.*
☐	☒	A description of all covariates tested
☒	☐	A description of any assumptions or corrections, such as tests of normality and adjustment for multiple comparisons
☐	☒	A full description of the statistical parameters including central tendency (e.g. means) or other basic estimates (e.g. regression coefficient) AND variation (e.g. standard deviation) or associated estimates of uncertainty (e.g. confidence intervals)
☐	☒	For null hypothesis testing, the test statistic (e.g. F, t, r) with confidence intervals, effect sizes, degrees of freedom and P value noted<br>*Give P values as exact values whenever suitable.*
☒	☐	For Bayesian analysis, information on the choice of priors and Markov chain Monte Carlo settings
☒	☐	For hierarchical and complex designs, identification of the appropriate level for tests and full reporting of outcomes
☒	☐	Estimates of effect sizes (e.g. Cohen's d, Pearson's r), indicating how they were calculated

Our web collection on statistics for biologists contains articles on many of the points above.

Software and code

Policy information about availability of computer code

Data collection	Observational data was recorded using a common spreadsheet. No special software was developed for collecting and storing data.
Data analysis	We have used standard and contributed MATLAB® (R2019a) functions or libraries to conduct experiments. In particular, we have used function lassoglm for logistic regression with elastic net regularisation, function TreeBagger for the random forest, and the Gaussian Process Regression and Classification Toolbox (version 4.2, gaussianprocess.org/gpml/code/matlab/doc/) for deriving GP models.

For manuscripts utilizing custom algorithms or software that are central to the research but not yet described in published literature, software must be made available to editors and reviewers. We strongly encourage code deposition in a community repository (e.g. GitHub). See the Nature Research guidelines for submitting code & software for further information.

Data

Policy information about availability of data

All manuscripts must include a data availability statement. This statement should provide the following information, where applicable:

- Accession codes, unique identifiers, or web links for publicly available datasets
- A list of figures that have associated raw data
- A description of any restrictions on data availability

The data sets generated during and/or analysed during the current study are not publicly available due to their sensitive nature and cannot be shared upon request as this would require specific new written consent from the parents of each child.

Field-specific reporting

Please select the one below that is the best fit for your research. If you are not sure, read the appropriate sections before making your selection.

- [ ] Life sciences
- [x] Behavioural & social sciences
- [ ] Ecological, evolutionary & environmental sciences

For a reference copy of the document with all sections, see nature.com/documents/nr-reporting-summary-flat.pdf

Behavioural & social sciences study design

All studies must disclose on these points even when the disclosure is negative.

Study description	In this quantitative study, we explore the potential of artificial intelligence as an approach in autism education to assist teachers in effective practice in developing social and educational outcomes for children with autism spectrum condition. We form a protocol to systematically capture such interactions, and conduct a statistical analysis to uncover basic patterns in the collected observations, including the longer-term effect of specific teacher communication strategies on student response. In addition, we deploy machine learning techniques to predict student response given the form of communication used by teachers under specific classroom conditions and in relation to specified student attributes.
Research sample	A data set was formed through structured classroom observations in 20 full-day sessions over 5 months in 2019 at a special school with criteria of ASC for admission in East London. Participants included 3 teachers (1 male, 2 females), their teaching assistants (all females), and 7 children (4 males, 3 females) aged from 6 to 12 years across 3 classes. The sample is fairly heterogeneous. Given the small amount of student participants (7), we note that it might not be representative. We also note that data collection in this context is quite challenging.
Sampling strategy	We used all the collected data in our analysis (no sampling has been carried out).
Data collection	A coding protocol was developed through an iterative process with the participating teachers, and a grid was used for recording teacher-student interaction observations. Comments and suggestions from the teachers were taken into consideration and reflected throughout the multiple revised drafts and the final versions of the coding protocol and recording grid. For each observation instance, we recorded the student identifier, time stamp, teaching objective, teaching type, context for this teaching type, student's observed emotional state, teacher's communication strategy, and the corresponding student response (outcome). Where applicable we also recorded additional notes and the type of activity (e.g. yoga). Although notes were used for context and interpretation for the data analysis as a whole, they were not included in our machine learning function experiments given their free-form inconsistency.
Timing	Teacher-student interactions were coded on particular dates in 2019. These were the following:<br>January 22, 29, and 30<br>February 5, 6, 12, 13, 26, and 27<br>March 12, 19, 22, 26, and 28<br>April 1, 2, and 25<br>May 1, 2, and 5
Data exclusions	No data was excluded.
Non-participation	No participants dropped out during the study.
Randomization	Wherever required (e.g. 10-fold cross-validation) student assignment to groups has been random.

Reporting for specific materials, systems and methods

We require information from authors about some types of materials, experimental systems and methods used in many studies. Here, indicate whether each material, system or method listed is relevant to your study. If you are not sure if a list item applies to your research, read the appropriate section before selecting a response.

Materials & experimental systems

n/a	Involved in the study	
x		Antibodies
x		Eukaryotic cell lines
x		Palaeontology and archaeology
x		Animals and other organisms
	x	Human research participants
x		Clinical data
x		Dual use research of concern

Methods

n/a	Involved in the study	
x		ChIP-seq
x		Flow cytometry
x		MRI-based neuroimaging

Human research participants

Policy information about studies involving human research participants

Population characteristics	See above.
Recruitment	We worked closely with the school's Outreach Officer who helped to share the participant recruitment information with interested teachers, parents and children. No self-selection biases that may impact results were present. We aimed to recruit children with a range of attributes (age, SCERTS, P-level). However, as the school acted as gatekeeper for selection of students, there may have been some unknown selection bias, but there is no reason to believe that this was systematic. We received signed consent forms from 7 parents, their children's class teachers, and the headteacher.
Ethics oversight	Ethical approval was granted by the Research Ethics Committee at the Institute of Education, University College London (United Kingdom).

Note that full information on the approval of the study protocol must also be provided in the manuscript.
